# A Novel Model Using Serum Thymidine Kinase 1 and Low-dose Computed Tomography Parameters to Predict Three-year Lung Cancer Risk in People with Pulmonary Nodules: A Retrospective Study

**DOI:** 10.7150/jca.90428

**Published:** 2024-01-01

**Authors:** Bei Yao, Xiaoyang Huang, Fei Wu, Jin Li, Ellen He, Xiaojing Li, Mukun Zhang, Ji Zhou, Haiou Hong, Sven Skog, Haidong Wang

**Affiliations:** 1 First Affiliated Hospital of University of Science and Technology of China, Division of Life Sciences and Medicine, University of Science and Technology of China, Hefei, 230001, China.; 2 Department of Medicine, Shenzhen Ellen-Sven Precision Medicine Institute, Shenzhen, 518000, China.; 3 China Health Promotion Foundation, Beijing, 100161, China.

## Abstract

This study was designed to develop a model of serum thymidine kinase 1 protein (STK1p) concentration in combination with low-dose computed tomography (LDCT) to predict the risk of benign pulmonary nodules progressing into lung cancer within three years in a large screening population. The study included a retrospective cohort of 6,841 individuals aged > 30 years who had LDCT-detected pulmonary nodules, but no cancer history or baseline cancer. The outcome was a lung cancer diagnosis recorded within three years after the first detection of pulmonary nodules. The adaptive least absolute shrinkage and selection operator was used to select candidate predictors and fit a logistic model. The model was validated internally by examining discrimination (area under the receiver operating characteristic curve (AUC), calibration (calibration plot)) and net benefit. A web application was developed based on the model. The results showed that the proportion of incident lung cancer cases was 0.79% (n=52). Predictors selected for the model were STK1p and three LDCT parameters (nodule size, type, and count). The AUC of the model was 0.91 (95% confidence interval (CI): 0.86, 0.96). The model had satisfactory discrimination at internal validation (AUC: 0.90 (0.84, 0.96)) and in subgroups (AUC=0.69-0.93). The high-risk group identified by the model exhibited a significantly higher three-year lung cancer risk than the low-risk group (odds ratio (OR): 66.03 (95% CI: 30.49, 162.98)). We concluded that the novel model of STK1p and LDCT parameters together can be used to accurately predict the three-year risk of lung cancer in people with pulmonary nodules.

## Introduction

Lung cancer is a major contributor to the burden of global health, with an estimated 1.8 million deaths worldwide in 2020 1. In China, lung cancer had an estimated 0.7 million deaths in 2020 2. It is essential to detect and treat the disease in its early stages. Low-dose computed tomography (LDCT) scans have been demonstrated to be an effective way to identify lung cancer in its early stage and reduce mortality. However, a challenge of using LDCT is assessing the lung cancer risk of pulmonary nodules. The false-positive rate was as high as 23.3% at the two-year follow-up test 3. This can result in unnecessary radiation exposure, invasive biopsies, financial costs, and patient anxiety due to further follow-up tests 4. It is necessary to improve risk assessment for pulmonary nodules following LDCT tests to recommend optimal follow-up strategies.

Current lung cancer screening guidelines in China recommend further examinations solely based on the size and density of pulmonary nodules 5. Meanwhile, European and British guidelines have suggested using prediction models that significantly reduce the false-positive rate 6,7. Published models for Chinese populations are generally based on a small sample size (<1000), which limits their generalizability 8-10. Models assessing the risk of lung cancer progression for Chinese populations with pulmonary nodules require further investigation. Despite the detection of small pulmonary nodules by LDCT scans, the risk of lung cancer progression in a person still exists. Finding and validating serum biomarkers for early lung cancer detection might be a key challenge in combination with LDCT scans.

Serum biomarkers for early cancer detection should be able to detect very small signals of early cancer amid the noise of normal human biology. Many biomarkers for early cancer detection have been proposed based on antibodies against different cancers 11, but few have been validated in large screenings. For example, elevated PSA in the blood is a candidate biomarker for the early detection of prostate cancer. However, it varies widely between and within individuals as they age or as they develop other nonmalignant prostate diseases. Therefore, PSA is not generally recommended for population-level screenings 11. To the best of our knowledge, there is no validated biomarker for predicting the risk of lung cancer progression in noncancerous pulmonary nodules in large screenings.

Human thymidine kinase 1 (HTK1) was found to assess the proliferation rate of growing cells in the 1960s. It is a key enzyme involved in DNA synthesis during the cell cycle and is regarded as an S-phase-specific enzyme. The near C-terminal of 31-peptide-195GQPAG PDNKE NCPVP GKPGE AVAAR KLFAPQ225 in HTK1 is a critical sequence for cell cycle regulation 12,13. The development of chicken HTK1-IgY-polyclonal antibodies raised against the C-terminal of 31-peptide had high sensitivity and specificity in an enhanced chemiluminescence (ECL) immune-dot blot with a biotin-streptavidin platform for early cancer in large screens 13,14. To date, data from approximately 160,000 persons undergoing health screenings have been analyzed for serum thymidine kinase 1 protein (STK1p) using the ECL dot plot 13. Among them, 340 persons were followed for up to 11 years, indicating that people with high STK1p (>2.0 pmol/L) had a 3-5-fold increased risk of developing malignant tumors 15. The lowest STK1p concentration detected by the ECL dot blot was 0.01 pmol/L (≈ 0.96 pg/ml) based on the analytical procedure of the Limit of Blank (LoB) and Limit of Detection (LoD) 16. With high sensitivity, this ECL dot blot was found to be able to discover people at risk of developing premalignancies and invisible malignancies in serum 13, 16, while a mouse monoclonal antibody was not found to have this ability on a Sandwich ELISA 17.

In this retrospective study, we aimed to screen potential predictors, including STK1p and LDCT parameters, for predicting three-year lung cancer risk for people who had noncancerous pulmonary nodules in a large health screening population.

## Materials and Methods

### Study design

This retrospective study was designed to develop a novel model of STK1p in combination with LDCT scanning to predict the risk of benign pulmonary nodules progressing into lung cancer within three years in a large screening population. The STK1p values were detected by a highly sensitive and specific enhanced chemiluminescence (ECL) immune-dot blot with a biotin-streptavidin platform based on a chicken human TK1-IgY-polyclonal antibodies raised against the near C-terminal critical sequence of 31-peptide-^195^GQPAG PDNKE NCPVP GKPGE AVAAR KLFAPQ^225^ in human TK1 during the cell cycle regulation 13,14.

### Participates

**Figure 1:**
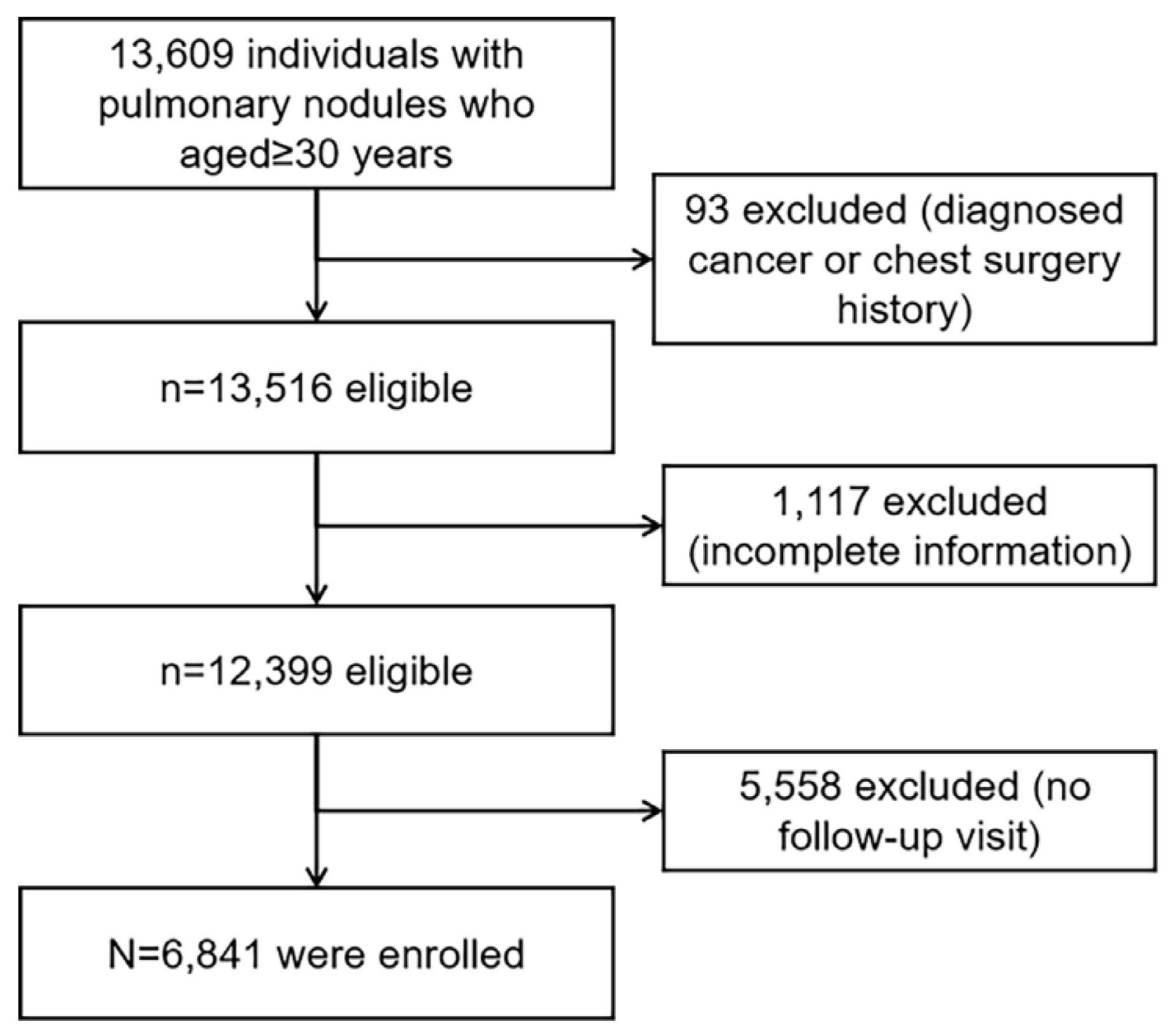
Study flowchart.

A retrospective cohort of 13,609 individuals was screened for eligibility (Figure 1). The cohort comprised individuals from the health management center of the First Affiliated Hospital of the University of Science and Technology of China in Anhui, China who attended health screenings, had LDCT-detected pulmonary nodules and were aged >30 years between January 1, 2018, and December 31, 2021. Among them, 6,841 participants (50.27%) met the eligibility criteria.

We derived anonymous patient data from the electronic medical records (EMRs) of the hospital. The baseline visit was defined as the visit with the first recorded pulmonary nodules. Individuals who had complete baseline information on candidate predictors were considered eligible. Individuals who had at least one follow-up visit were considered eligible. We excluded individuals with baseline cancer. Baseline cancer was defined by the presence of an International Classification of Diseases (ICD)-9 or ICD-10 code of any type of cancer prior to the baseline visit or a recorded cancer history. We also excluded those who had a history of thoracic surgery at baseline to avoid the inclusion of potential lung cancer cases.

### STK1p assay

STK1p was routinely measured at the health management center. The concentration of STK1p in serum is very low. Therefore, we used a highly sensitive detection assay. According to the principle of a highly sensitive biotin-streptavidin detection system, the ECL dot blot on the biotin-streptavidin platform was developed based on HTK1-IgY-polyclonal antibodies (Sino-Swed Tong Kang Bio-Tech Ltd., Shenzhen, China) against the C-terminal of the 31-peptide 13, 14. Briefly, three µl serum was dotted on a nitrocellulose membrane (Hyband^TM^ C, Cytiva, USA), incubated with primary biotinylated HTK1-IgY-polyclonal antibodies for 30 minutes, and then incubated with streptavidin-horseradish peroxidase (Invitrogen, Thermo Fisher Scientific, USA) for 30 minutes followed by the addition of ECL substrate (Beijing KEY-BIO Biotech Co., Ltd., China). The light intensity of a single spot on the membrane was detected using a CIS-II imaging system (Sino-Swed Tong Kang Bio-Tech (Shenzhen) Ltd., China) based on the intensity of the HTK1 standard of known concentrations. The intensity of STK1p in the serum samples was recalculated and expressed as pmol/L.

### Thoracic LDCT scans

Thoracic LDCT scans (Optima CT660, GE Healthcare, Japan) were routinely performed and analyzed by radiologists at the health management center of the hospital. Key LDCT parameters, including pulmonary nodule type, size and count, were validated by experienced radiologists before being entered into the EMRs.

### Predictors

We chose 13 baseline parameters that were routinely recorded by the EMRs as candidate predictors for the model. Details of these parameters are described as follows:

Age, sex, and smoking status were self-reported by the patient and recorded by physicians using EMRs. In the case of individuals without a record of smoking status, physicians' notes were further reviewed. Patients without information on smoking status were not included.

We identified three serum biomarkers, including STK1p, carcinoembryonic antigen (CEA), and alpha-fetoprotein (AFP), measured at the baseline visit. STK1p was measured by the ECL dot plot described above. The serum CEA and AFP levels were determined using chemiluminescence analyzers (ALINITY ci-series, Abbott, Illinois, USA). Information on these serum biomarkers in the EMRs was automatically linked to the laboratory.

We identified four clinical characteristics, including body mass index (BMI), hypertension, hyperglycemia, and dyslipidemia. BMI (kg/m^2^) was calculated based on self-reported height and weight. Hypertension was defined by the presence of a recorded diagnosis, a high level of systolic blood pressure (≥130 mmHg), or diastolic blood pressure (≥80 mmHg). Hyperglycemia was defined by the presence of a recorded diagnosis or a high level of fasting plasma glucose (≥6.1 mmol/L) or two-hour plasma glucose (≥7.8 mmol/L). Dyslipidemia was defined by the presence of a recorded diagnosis or an abnormal level of total cholesterol (≥6.2 mmol/L), triglycerides (≥11.3 mmol/L), low-density lipoprotein cholesterol (≥4.14 mmol/L), or high-density lipoprotein cholesterol (<1.04 mmol/L).

We identified three parameters, including pulmonary nodule type, size and count, from the recorded LDCT reports. Nodule type and size were obtained for a single nodule or the nodule with the largest diameter when a participant had more than one nodule. Nodule type (solid or subsolid (including part-solid or ground-glass)) was defined based on the density of the nodule. Nodule size (mm) was defined as the largest diameter of the solid area for part-solid and solid nodules or the largest diameter for ground-glass nodules. We also obtained information on nodule count (multiple or single). These LDCT parameters were routinely recorded and validated by experienced radiologists.

### Outcomes

The outcome of the model was incident lung cancer occurring within three years after the baseline visit. The occurrence of lung cancer was defined as the recorded specialist-diagnosed lung cancer using ICD-9 and ICD-10 codes through the Hospital Episode Statistics system. Individuals without a code of incident lung cancer were assumed to be lung cancer-free. It was not possible to contact individuals to confirm whether they were diagnosed with lung cancer in other hospitals because of ethical restrictions. For participants with the outcome, the study endpoint was the date when the outcome was recorded. For participants without the outcome, the study endpoint was the final visit date within three years after the baseline visit.

### Statistical analysis

Medians with interquartile ranges (IQRs) were calculated for continuous variables. Pearson's Chi-squared test (for expected cell count of five or more) or Fisher's exact test (for expected cell count less than five) was used to compare categorical variables, and the Wilcoxon rank-sum test was used to compare continuous variables between groups with and without incident lung cancer.

The adaptive least absolute shrinkage and selection operator (LASSO) was used to select candidate predictors and fit a logistic regression model 18. Odds ratios (ORs), their 95% confidence intervals (CIs) and p values were estimated for each selected predictor.

The prediction performance was evaluated by discrimination (ability to classify accurately) and calibration (whether model-estimated probabilities are equal to observed probabilities). Discrimination was measured by the area under the receiver operating characteristic (ROC) curve (AUC) and its 95% CI. Calibration was measured by the ratio between the model's expected probability and observed probability (E:O) of lung cancer within three years, calibration-in-the-large (CITL), and calibration slope. A calibration plot was used to visually present the model's predicted probability versus observed probability. The model was internally validated using tenfold cross-validation. Subgroup analyses based on categorical variables were performed to further evaluate the model performance.

The optimal threshold of the model-predicted probability was determined using the Youden Index (sensitivity+specificity-1), a metric that evaluates the trade-off between sensitivity and specificity. Net benefit was used to calculate a weighted sum of true- minus false-positive classifications at a threshold. It facilitates the clinical judgment of the relative value of benefits (such as detecting cancer) and harms (such as unnecessary tests) associated with a model 19. Decision curves were used to present the net benefit for the model and each selected predictor by different threshold probabilities.

All statistical analyses were performed, and figures were made using R version 4.2.2 and Stata/MP version 17.0. All statistical tests were two-sided, and a p value of less than 0.05 was considered significant.

## Results

### Study participants

The number of excluded individuals was 6,768 (49.73%): 93 (0.68%) had a baseline cancer or thoracic surgery history, 1,117 (8.21%) had incomplete baseline information, and 5,558 (40.84%) had no follow-up visit. The median age (51 (IQR 45-59) versus 53 (45-61) years) and the proportion of females (40.32% versus 39.74%) of the 6,841 eligible participants were similar to those of the 13,609 individuals who were screened. The median follow-up of the study participants was 3.00 years (IQR: 3.00-3.40). The ages of the participants ranged from 31 to 91 years. During the follow-up, 52 (0.76%) participants had incident lung cancer. Baseline characteristics of age, sex, smoking, and clinical characteristics were similar between participants with and without incident lung cancer (Table 1). The baseline proportion of participants with subsolid nodules (p<0.001) and multiple nodules (p<0.001) was higher in participants with incident lung cancer than in those without. The baseline nodule size (p<0.001) and STK1p (p<0.001) were also higher in participants with incident lung cancer than in those without. There was no significant difference in other baseline characteristics between participants with and without incident lung cancer.

### Predictive model

**Figure 2:**
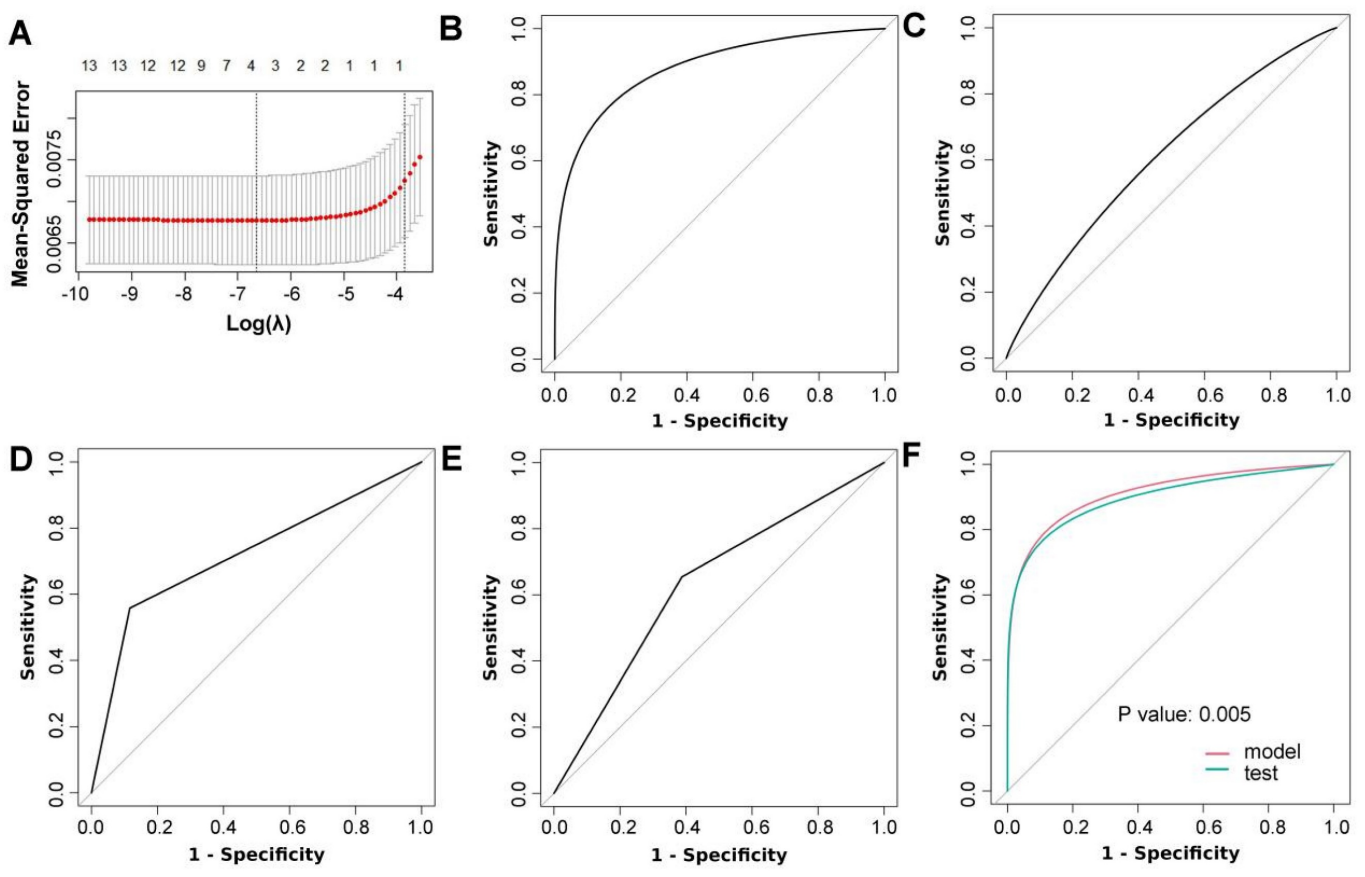
Predictor selection and validation of the model. (A) Adaptive least absolute shrinkage and selection operator (LASSO) results; receiver operating characteristic (ROC) curves for nodule size (B), serum thymidine kinase 1 protein (C), nodule type (D) and nodule count (E); (F) ROC curves for the 3-year lung cancer risk model and internal validation (test) (DeLong's test was used to compare two ROC curves).

**Figure 3:**
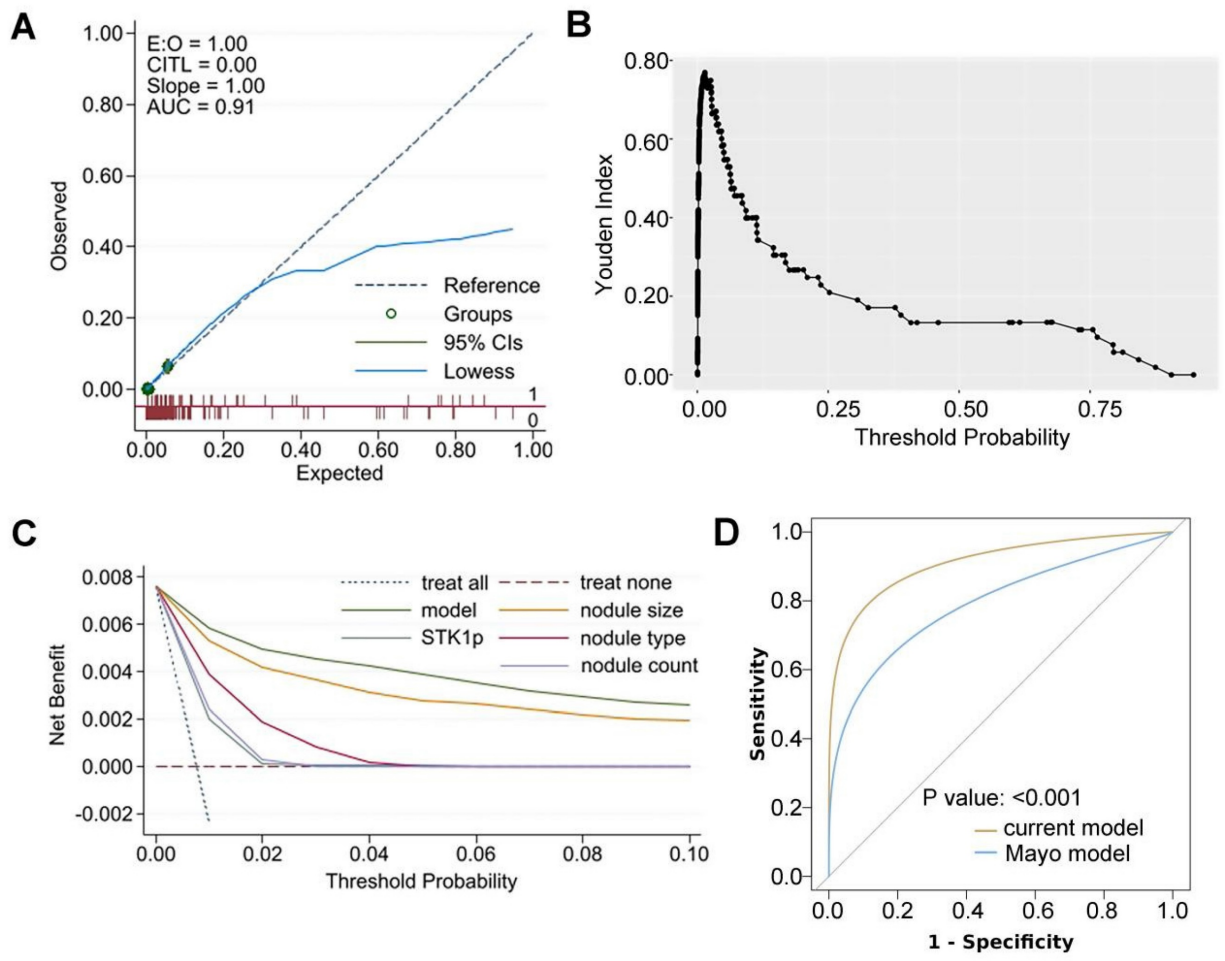
(A) calibration plots for the model expected lung cancer probability and observed probability; (B) Youden index by the model expected probability; (C) decision curves for the model and each predictor applied in the study participants; (D) receiver operating characteristic (ROC) curves for the current model and the Mayo model in a subset of study participants (DeLong's test was used to compare two ROC curves); E:O: expected versus observed probability of 3-year lung cancer; CITL: calibration-in-the-large; AUC: area under receiver operating characteristic curve; STK1p: serum thymidine kinase 1 protein.

The LASSO procedure was used to select nodule size, nodule type (subsolid versus solid), nodule count (multiple versus single) and STK1p for the logistic model (Figure 2A; Supplementary Table 2). Univariate and multivariate logistic models showed that each predictor was associated with three-year lung cancer (Table 2). Collinearity tests showed that there was no likely correlation between a given predictor and any other predictors in the model (Supplementary Table 2). Model coefficients are presented in Table 3. The AUC of the model was 0.91 (95% CI: 0.86, 0.96) and was higher than that of each predictor (Figure 2B-F; Table 4). The calibration plot showed that the model expected probabilities of lung cancer were close to the observed probabilities in participants who had expected probabilities under 0.4 (Figure 3A). The probabilities of lung cancer were underestimated by the model in a small group of participants who had expected probabilities over 0.4.

### Validation of the model

The model AUC (0.90 (95% CI: 0.84-0.96)) (Figure 2F) was also excellent for internal validation. The model AUC was satisfactory in subgroups (ranging from 0.69-0.93), even for nodules smaller than 6 mm (0.69) (Supplementary Table 3). The model calibration statistics were generally adequate in most subgroups.

However, in participants with part-solid nodules, the three-year lung cancer risk was likely to be underestimated by 32% (E:O: 0.68) by the model. This was likely due to the small number of lung cancer cases in this group (n=8).

The optimal threshold of the model predicted probability (Youden index=76.90%) was 0.02 (Figure 3B). The high-risk group (model predicted probability ≥0.02) exhibited a significantly higher three-year lung cancer risk than the low-risk group (odds ratio (OR): 66.03 (95% CI: 30.49, 162.98); p<0.001). The sensitivity and specificity at the optimal threshold were 84.60% (a false-negative rate of 15.40%) and 92.30% (a false-positive rate of 7.70%), respectively. These were higher than the sensitivity and specificity at the optimal threshold of individual predictors (Table 4). Decision curves for the model and individual predictors are shown in Figure 3C. Assuming participants who have a probability over the threshold are offered follow-up tests (such as positron emission tomography (PET)-computed tomography (CT)), the net benefit is consistently higher when testing based on the model than when testing all participants or testing based on individual predictors (Figure 3C).

In addition, an established model for prevalent lung cancer, the Mayo model for solid nodules 20, was tested using a subset of 3,720 (54%) study participants who had complete data of the model's predictors and met the model's inclusion criteria (Supplementary Table S4). Our model showed a significantly higher AUC (0.87) than the Mayo model (0.76) (p<0.001), as presented in Figure 3D.

### Web application

**Figure 4:**
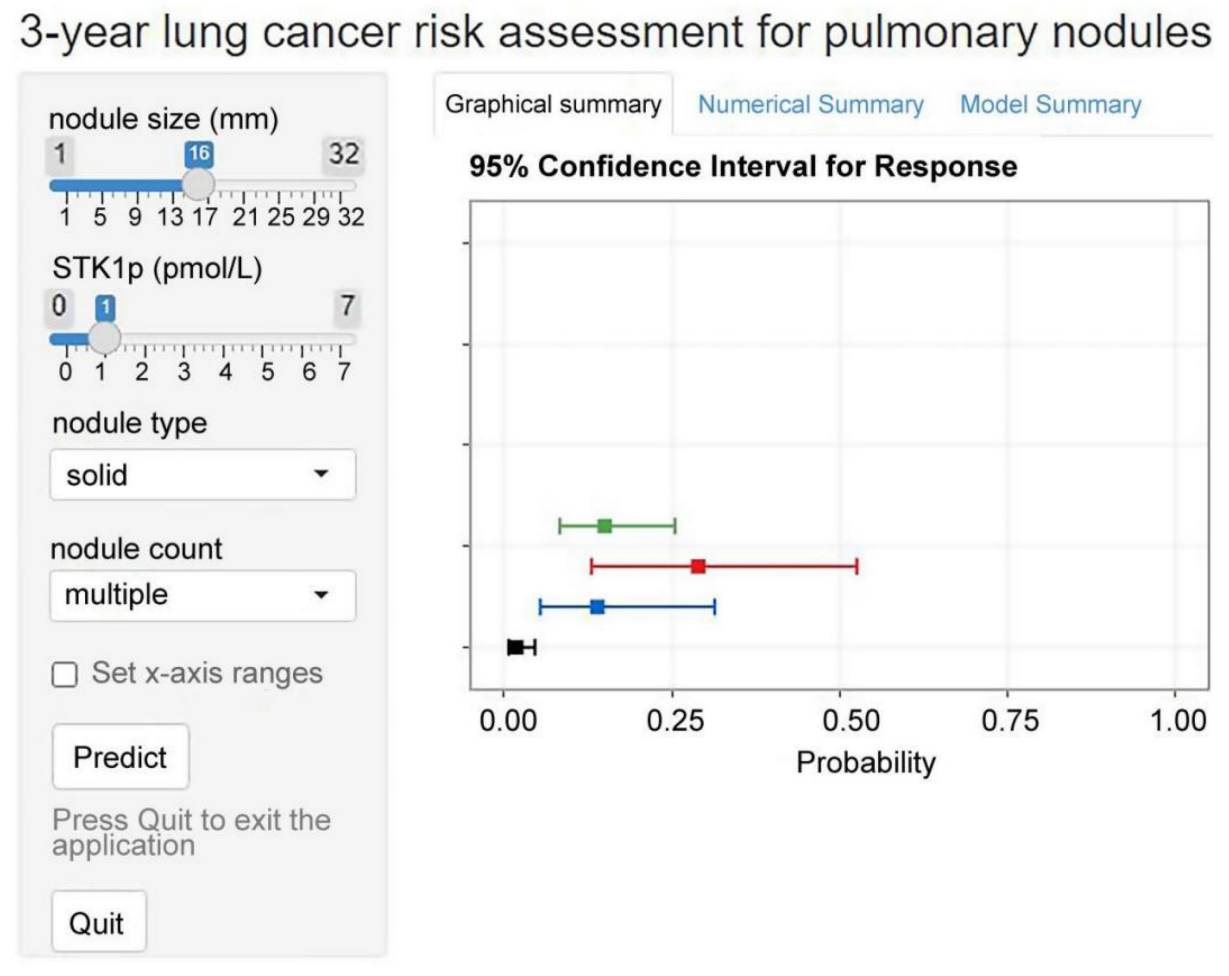
Example of results from the web application of the model.

We implemented the model into a web application that offers risk predictions for individuals with pulmonary nodules based on input predictors. It can calculate the probability of 3-year lung cancer and present it visually. The web application was made accessible online (Figure 4).

## Discussion

This retrospective study of a Chinese population developed a novel model predicting the probability of noncancerous pulmonary nodules first detected by LDCT becoming lung cancer within three years. The model incorporates LDCT parameters, including nodule size, type and count, and a novel predictor, STK1p. The model shows excellent predictive accuracy, with an AUC of 0.91. The model discrimination and calibration were validated internally and in subgroups, even for nodules smaller than 6 mm (AUC: 0.76). If a threshold of 0.02 was used for the model-predicted probability of lung cancer, the sensitivity and specificity were 84.60% (a false-negative rate of 15.40%) and 92.30% (a false-positive rate of 7.70%), respectively.

The results of previous studies have indicated that imaging features provide valuable information on the pathological characteristics of pulmonary nodules, which is in line with our findings indicating that LDCT-based nodule size, type, and count were significantly associated with three-year lung cancer risk 6,7,21,22. In contrast with previous literature 21-23, our findings did not show a significant association between cancer-related biomarkers such as CEA and incident lung cancer, perhaps because of the small number of cases with incident lung cancer. As tracking individuals in person was not available in this study, it was impossible to identify lung cancer cases diagnosed in other hospitals or lung cancer deaths that occurred in non-hospital settings. We will propose a prospective cohort study with regular follow-ups to comprehensively identify incident lung cancer cases and to further assess the significance level of tumor biomarkers in lung cancer risk prediction.

Our model includes a novel predictor, STK1p, that is less invasive, low-cost, and easily obtained. It has been reported that STK1p is sensitive to the presence of various types of cancer 24, such as lung cancer 25, colorectal cancer 26,27, and prostate cancer 28. This biomarker has also shown predictive value for various types of cancer, including lung cancer 29. This study highlights the potential of STK1p to improve lung cancer risk prediction.

Compared with previous similar models, our model is a combination of STK1p and LDCT, which is the first time to incident for predicting the risk of benign pulmonary nodules progressing to lung cancer, while a previous study focused on the diagnostic model of STK1p combined with spectral dual-layer computed tomography scanning for a diagnosis of patients with invasive ground glass nodules 30. Therefore, we are the first to identify subgroups of patients with newly detected benign pulmonary nodules who are more likely to develop lung cancer within three years. Our results will then inform follow-up strategies for these patients. Additionally, our model only requires STK1p, a reliable tumor proliferating biomarker, in combination with LDCT parameters that are easily obtained, while most models require self-reported data (e.g., smoking pack years, family history), LDCT parameters, and multiple serum biomarkers (e.g., STK1, CYFRA21-1, miRNA). Thus, compared to previous research, our study is distinctive by providing a convenient and cost-effective approach, especially for early lung cancer prediction in large-scale screenings. Compared with previous studies for lung cancer risk prediction in people with pulmonary nodules, our model also has advantages in sample representativeness. We identified a large sample of individuals with pulmonary nodules detected at health screenings with a wide range of sample ages (31-91 years) and no criteria on nodule characteristics. Therefore, our study findings are more likely to be generalized to the real-world population with pulmonary nodules compared with previous studies that focused on specific nodule types.

British and European guidelines have recommended using prediction models to facilitate clinical decision-making in the management of pulmonary nodules 6,7. In China, there is little evidence of risk prediction models for pulmonary nodules, and current guidelines have not included prediction models to date. Therefore, this study has important clinical implications. This model has the potential to aid clinical decision-making, such as offering PET-CT for patients with pulmonary nodules with a high predicted risk, even in cases of smaller nodules (<6 mm). To enable the implementation of the model in screening practice, we integrated our findings into a web application, which provides a convenient assessment of lung cancer risk. However, these tools still require further study and external validation. At present, they should not displace the well-informed clinical judgment of physicians or be applied as a complementary tool for treatment plan decision-making.

The major limitation of the study is the considerable loss of follow-up; 45% of eligible individuals had no follow-up visit. This might result in missing incident lung cancer cases and underestimating lung cancer risk. As stated above, we will propose a prospective cohort study to address this issue. A larger number of lung cancer cases is expected to be used in developing models in subgroups based on key parameters, such as those with smaller nodules that were not allowed in the current study. However, the model was validated in subgroups and showed satisfactory performance. Furthermore, the model lacks information on other potential predictors of lung cancer, such as cytokeratin 19 fragment (CYFRA 21-1) and squamous cell carcinoma antigen because these predictors were not routinely collected in the study setting. Therefore, we will validate and update the model in future studies by planning regular follow-up visits, extending the follow-up time, ensuring the recording of nodule size, and collecting more data on other predictors. The cost-effectiveness of different models will also be measured in a future study to help select the optimal model.

## Conclusions

This novel model is the first to use STK1p, a reliable tumor proliferating serum biomarker, combined with LDCT parameters to predict the risk of pulmonary nodules progressing into lung cancer within three years in a large screening population. The implementation of our model and coupled web application is expected to enable accurate prediction of lung cancer risk to guide additional tests or follow-up strategies in people with pulmonary nodules. Future prospective research is needed to externally validate and update the model.

## Supplementary Material

Supplementary tables.

## Figures and Tables

**Table 1:** Baseline characteristics of 6,841 study participants

Characteristic			No incident lung cancer (n=6,789)	Incident lung cancer (n=52)	P
Age (year)			51.00 (45.00, 59.00)	52.5(48.75, 59.00)	0.19
Sex					0.77
	Female		2,736 (40.30)	22 (42.31)	
	Male		4,053 (59.70)	30 (57.69)	
Nodule size (mm)			4 (3, 5)	12 (7, 17)	**<0.001**
Nodule type					**<0.001**
	Solid		6,000 (88.38)	23 (44.23)	
	Subsolid	Ground-glass	605 (8.91)	21 (40.38)	
		Part-solid	184 (2.71)	8 (15.38)	
Nodule count					**<0.001**
	Single		4,157 (61.23)	18 (34.62)	
	Multiple		2,632 (38.77)	34 (65.38)	
Smoking			747 (11.00)	3 (5.77)	0.23
Serum biomarkers					
	STK1p (pmol/L)		0.12 (0.10, 0.25)	0.24 (0.10, 0.38)	**<0.001**
	AFP (μg/L)		2.98 (2.21, 4.03)	2.86 (2.08, 3.97)	0.46
	CEA (μg/L)		1.57 (1.01, 2.35)	1.79 (1.01, 2.37)	0.42
Clinical characters					
	Body mass index (kg/m^2^)		26.08 (24.80, 27.76)	26.18 (25.43, 27.23)	0.44
	Hypertension		5,551 (81.76)	39 (75.00)	0.21
	Hyperglycemia		843 (12.42)	4 (7.69)	0.30
	Dyslipidemia		1,913 (28.18)	15 (28.85)	0.92

Data are median (interquartile range (IQR)) or n (%); STK1p: serum thymidine kinase 1 protein; AFP: alpha-fetoprotein; CEA: carcinoembryonic antigen; values in **bold** are statistically significant

**Table 2:** Results of univariate and multivariate logistic regression

Predictor	Univariate			Multivariate		
	Odds Ratio	95% CI	P	Odds Ratio	95% CI	P
Nodule size (mm)	1.40	1.33, 1.47	**<0.001**	1.36	1.30, 1.44	**<0.001**
STK1p (pmol/L)	1.44	1.04, 1.81	**0.009**	1.52	1.05, 1.98	**0.007**
Nodule type, subsolid vs. solid	9.59	5.53, 16.81	**<0.001**	6.75	3.60, 12.79	**<0.001**
Nodule count, multiple vs. single	2.98	1.70, 5.40	**<0.001**	2.52	1.33, 4.94	**0.006**

STK1p: serum thymidine kinase 1 protein; CI: Confidence Interval; values in **bold** are statistically significant

**Table 3:** Coefficients of the 3-year lung cancer risk model

Predictor	Coefficient	Standard error	Z	P
Nodule size (mm)	0.3090	0.0263	11.75	**<0.001**
STK1p (pmol/L)	0.4178	0.1549	2.70	**0.007**
Nodule type, subsolid vs. solid	1.9089	0.3213	5.94	**<0.001**
Nodule count, multiple vs. single	0.9234	0.3332	2.77	**0.006**
Intercept	-8.0186	0.4025	-19.92	**<0.001**

STK1p: serum thymidine kinase 1 protein; values in **bold** are statistically significant

**Table 4:** Optimal thresholds for the model expected lung cancer probability and individual predictors

Predictor	AUC (95% CI)	Threshold	Sensitivity	Specificity	Likelihood ratio (+)
Model	0.91 (0.86, 0.96)	≥0.02	84.60%	92.30%	10.99
Nodule size (mm)	0.87 (0.80, 0.94)	≥6	80.80%	86.70%	6.08
Nodule type, subsolid vs. solid	0.72 (0.65, 0.79)	≥1 (subsolid)	55.80%	88.40%	4.81
Nodule count, multiple vs. single	0.63 (0.57, 0.70)	≥1 (multiple)	65.40%	61.20%	1.69
STK1p (pmol/L)	0.64 (0.57, 0.72)	≥0.20	61.50%	66.80%	1.85

STK1p: serum thymidine kinase 1 protein; AUC: area under the receiver operating characteristic curve

## Abbreviations

AFP: alpha-fetoprotein
AUC: area under the receiver operating characteristic curve
CEA: carcinoembryonic antigen
CI: confidence interval
CITL: calibration-in-the-large
CT: computed tomography
EMR: electronic medical record
ICD: International Classification of Diseases
IQR: interquartile range
LASSO: least absolute shrinkage and selection operator
LDCT: low-dose computed tomography
OR: odds ratio
PET: positron emission tomography
ROC: receiver operating characteristic
STK1p: serum thymidine kinase 1 protein
