# Supplementary material for: A Novel Model Using Serum Thymidine Kinase 1 and Low-dose Computed Tomography Parameters to Predict Three-year Lung Cancer Risk in People with Pulmonary Nodules: A Retrospective Study
Source: J Cancer. 2024 Jan 1;15(3):737–46. doi: 10.7150/jca.90428 (PMC10777045; doi:10.7150/jca.90428)
Supplement: Supplementary file 1 — Supplementary tables. [file jcav15p0737s1.pdf]

| Table S1. Lasso estimation. | | | |
|---|---|---|---|
| | Lambda | Cross-validation deviance | Variables added |
| | 0.0139114 | 0.0833110 | Nodule size |
| | 0.0079606 | 0.0718943 | Nodule type |
| | 0.0028609 | 0.0651677 | STK1p |
| * | 0.0010281 | 0.0622002 | Nodule count |
| | 0.0008536 | 0.0623281 | AFP |
| | 0.0007087 | 0.0625902 | Hypertension |
| * lambda selected by cross-validation; STK1p=serum thymidine kinase 1 protein; AFP=Alpha-fetoprotein | | | |

| Table S2. Collinearity statistics. | | |
|---|---|---|
| Predictor | Variance inflation factor | Tolerance |
| Nodule size (mm) | 1.0019 | 0.9981 |
| Serum thymidine kinase 1 protein (pmol/L) | 1.0093 | 0.9908 |
| Nodule type, sub-solid vs. solid | 1.0072 | 0.9928 |
| Nodule count, multiple vs. single | 1.0064 | 0.9936 |

| Table S3. Performance of the prediction model in subgroups. | | | | | |
|---|---|---|---|---|---|
| Subgroup | | AUC | E:O | CITL | Calibration slope |
| Nodule size (mm) | ≥6 | 0.90 | 1.00 | 0.00 | 1.03 |
| | <6 | 0.69 | 1.00 | 0.00 | 0.84 |
| Nodule type | Ground-glass | 0.90 | 1.12 | -0.13 | 1.42 |
| | Part-solid | 0.84 | 0.68 | 0.47 | 0.84 |
| | Solid | 0.92 | 1.00 | 0.00 | 0.94 |
| Nodule count | Multiple | 0.87 | 1.00 | 0.00 | 0.94 |
| | Single | 0.93 | 1.00 | 0.00 | 1.10 |
| *E:O=expected versus observed probability of 3-year lung cancer; CITL=calibration-in-the-large; AUC=area under receiver operating characteristic curve | | | | | |

Table S4. Comparison of AUCs between the Mayo model and current model in the study participants.

| Model | Inclusion criteria | Outcome | Algorithms | AUC | *P |
|---|---|---|---|---|---|
| Current model | No cancer history or baseline cancer | Incident lung cancer | Lp=-8.0186 + 0.3090 × size (mm) + 1.9089 × type + 0.9234 × count + 0.4178 × STK1p (pmol/L) | 0.87 | Reference |
| Mayo model | Solid nodule, no cancer history within 5 years prior to baseline | Prevalent lung cancer | Lp= −6.8272 + 0.0391 × age (year) + 0.7917 × smoking + 1.3388 × cancer history + 0.1274 × size (mm) + 1.0407 × spiculation + 0.7838 × the upper lobe | 0.76 | <0.001 |

AUC=area under receiver operating characteristic curve; STK1p=serum thymidine kinase 1 protein; Lp=linear predictor; probability of lung cancer = exp(lp)/(1+exp(lp)); *DeLong's test was used to compare two AUCs
